# Case of Ulcerated Cæcum

**Published:** 1835-01-01

**Authors:** F. E. Hicks


*To the Editor of the Medical Quarterly Review.*

Sir: If you think the following case worth a place in your valuable journal, will you do me the favour to insert it in the next number? I am your obedient servant,

F. E. Hicks,
Late House Surgeon to St. George's Hospital.

33, *George Street, Hanover Square;*
*Dec.* 13, 1834.

On Saturday, the 6th inst. at 2 o'clock P.M. I was requested to visit a young lady, aged twenty. I was from home at the time, and did not see her until four o'clock on the same day.

I then learnt that, owing to a cold, she had remained in bed longer than usual that morning; that upon getting out of bed, a sudden and involuntary discharge of fæces had taken place, mixed with blood; I should think that it was nearly a pint in quantity, and apparently venous. A medical practitioner had seen and prescribed for her. She did not complain to me of any pain, but said that she felt restless and uneasy. Her countenance was pale, and the pulse was quick, feeble, and fluttering. Believing the bleeding to come from some of the hæmorrhoidal vessels, I examined the rectum, but could not satisfy myself that it did so. I, however, prescribed a cold astringent injection, and the following draught: ℞. Infusi Rosæ c. ℥iss. Acidi sulphurici diluti, m. xx. Conf. Rosæ Gall. ʒss. M. fiat haustus statim sumendus et omni horâ repetendus.

As she was placing herself in a position for the nurse to administer the injection, about two pints of blood were again expelled.

I was again sent for to see her about half-past five o'clock. She was faint; there was no pulse at the wrist; and the extremities were cold. I ordered the injection to be repeated, and gave her half an ounce of Ruspini's Styptic by the mouth. Several large coagula were again expelled from the rectum in the course of a few minutes. The syncope increased to such an alarming extent that I was obliged to give stimulants, and repeated the Ruspini's Styptic. I then requested my father to see her. The hæmorrhage did not recur after six o'clock.

We continued to give stimulants, but she never rallied from the state of syncope, and died about half-past seven o'clock in the evening.

On Monday, the 8th inst. I examined the body. The intestinal canal from the cæcum downwards was of a dark colour, and appeared to contain blood. On laying open the cæcum and upper portion of the colon I discovered a great number of circular ulcers, particularly about the valve of the cæcum, some of them as large as a sixpence. In the centre of most of them was seen a thick dark-coloured slough, which had separated at the edges; from some of them the slough had been entirely thrown off, leaving an unhealthy-looking ulcer. The cæcum and colon contained about half a pint of blood. The other viscera were healthy.

*History.* This young lady, for the last two years had suffered occasionally from dyspepsia and constipation of the bowels; but her ailments were seldom so severe as to require medical advice, the symptoms being generally relieved by a little aperient medicine. She does not appear to have complained of any pain in the abdomen.

About a week before her death she took cold, and her bowels became constipated, requiring strong and repeated doses of aperient medicine, prescribed by herself; such as castor oil, antibilious pills, &c.; these all failed until the day before her death, when the bowels were well relieved by a black draught. The following morning she felt much better; and indeed, when I saw her and made enquiries as to her previous state of health, she told me, that independently of the cold, she never imagined herself in better health.

*Remarks.* The extraordinary features of this case are, first: that it is clear the cæcum must have been a seat of disease for some time past, although the symptoms were never referred to this viscus; and, secondly, that so profuse an hæmorrhage should have taken place from these small ulcers as to destroy life.

I imagine, myself, that these ulcers had existed for a considerable length of time; that owing to the cold, probably attended with some fever, and owing to the irritation produced by the aperient medicines the patient had taken, they had put on a sloughing character; and that on the separation of the sloughs the hæmorrhage ensued. I could not discover any larger vessel into which the ulceration had extended; neither do I believe that bleeding to such an extent could take place from any one vessel of the cæcum or colon. I think then that this enormous loss of blood (altogether three quarts) was occasioned by nearly all these ulcers bleeding simultaneously.

